# Status of waste disposal of sharps outside medical institutions for patients with diabetes: A systematic review

**DOI:** 10.1371/journal.pone.0288993

**Published:** 2023-11-17

**Authors:** Jingwen Wu, Min Wang, Hong Yan

**Affiliations:** Nursing College of Chengdu University of Traditional Chinese Medicine, Twelve Bridge Campus of Chengdu University of Traditional Chinese Medicine, Jinniu District, Chengdu, Sichuan, People’s Republic of China; Wachemo University, ETHIOPIA

## Abstract

**Objectives:**

As the number of people with diabetes increases, so does the amount of household-generated sharp waste, and incorrect sharp disposal methods can expose the public to needle stick injuries This systematic study assesses the relevant factors and current situation of the disposal of sharp waste in diabetes patients.

**Methods:**

In this review, our study comprehensively searched PubMed, MEDLINE, Cochrane Library, EMBASE, Web of Science, and China Biomedical, Wanfang, and CNKI for the concepts of "sharps waste disposal" and "diabetes".

**Result:**

In 12 identified articles, there are 4155 patients with diabetes. The findings highlight that diabetic patients have a positive attitude towards sharps waste disposal, but lack knowledge and practice of sharps waste disposal, and need to take appropriate measures to improve the rate of proper waste disposal before and during use. Patients with longer duration of diabetes are more likely to engage in inappropriate sharps disposal behaviors.

**Conclusions:**

The findings emphasize that the majority of diabetic patients are unable to handle sharps safely, so more research is needed to find factors associated with sharps waste disposal in diabetic patients and to focus on sharps waste disposal behaviors in patients with longer duration of disease in future clinical practice.

**Trial registration:**

**PROSPERO ID.** The review was registered on PROSPERO (registration number: CRD42023427592) https://www.crd.york.ac.uk/prospero/display_record.php?ID=CRD42023427592.

## Introduction

Approximately 422 million people worldwide have diabetes mellitus (DM), and its prevalence has been increasing over the past decades [1]. The World Health Organization defines sharps as "objects that can cause cuts or puncture wounds, including needles, hypodermic syringes, scalpels, etc [2]. It is estimated that nearly 29% of people with diabetes require insulin to control their blood glucose levels, and maintaining optimal glycemic control usually requires patients to perform self-monitoring of blood glucose (SMBG) [3–5]. More than 2 billion needles and syringes are used annually for self-injection in patients with diabetes [6]. However, sharps handling in the home setting is less studied and less addressed, resulting in inconsistent approaches to proper sharps use, which increases the risk of needlestick injuries and infections in the home [7–9].

Historically, most of the public health consequences of diabetes have been described as complications and economic burdens [10], but the environmental impacts and public health risks associated with unsafe disposal of sharps arising from diabetes self-management have been rarely discussed [5,11]. Proper disposal of medical sharps as part of patient education on self-injection techniques is often overlooked, leading to potentially unsafe disposal practices [12–15]. Improper disposal of sharps waste not only causes environmental contamination [16], but also poses a great danger to all those who inadvertently dispose of common household waste [17]. For example, not only workers at material recovery facilities, but also landfill workers and those who collect household waste are exposed to considerable risks. [18,19] Each year, approximately 5.2 million people worldwide die from diseases caused by improper sharps waste management [20]. Improper disposal of sharps can lead to the spread of several diseases such as hepatitis B, hepatitis C, and AIDS, and put people who come in contact with these sharps at risk [21–24]. This can become a serious public health problem because these diseases are chronic infections that often go undetected for a long time [25].

As far as we know, there are no systematic studies to evaluate diabetic patients for sharps waste disposal. The purpose of this study was to collect all available data, analyze the factors affecting it, and understand the knowledge, practices, and attitudes of diabetic patients regarding sharps waste disposal in order to provide valuable scientific information to health care decision makers.

## Material and methods

**Fig 1:**
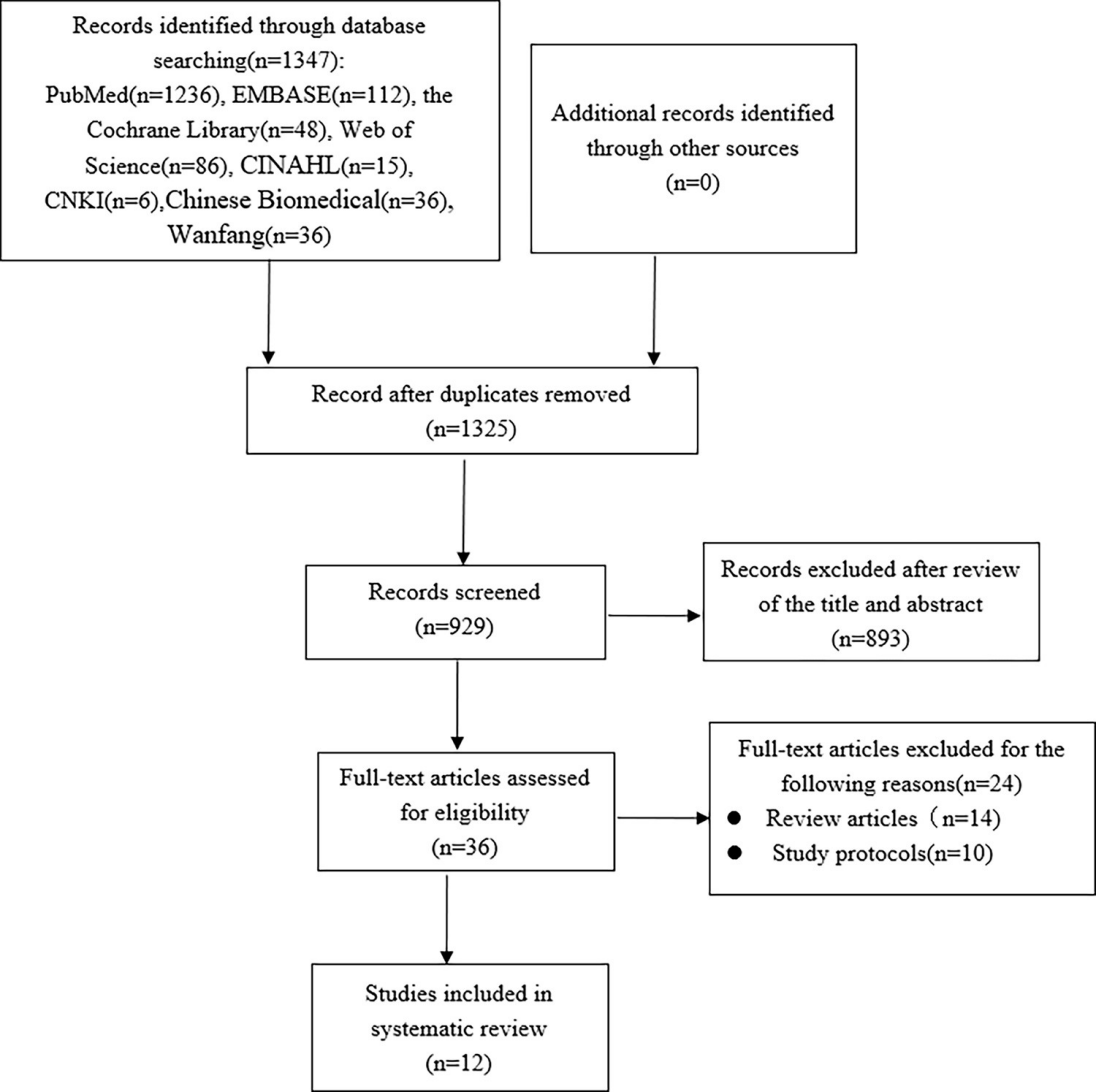
Flow diagram for search and selection of the included studies.

After a database search, a total of 1347 possible items were identified, as shown in Fig 1. Of these projects, 1325 were reused and subsequently excluded. After preliminary screening of the remaining article abstracts, it was determined that 929 articles did not meet the inclusion/exclusion criteria. A total of 36 articles were identified for full text review. Only 12 of these 56 studies met the necessary inclusion/exclusion criteria. Systematic review were performed according to (PRISMA) Preferred Reporting Items for Systematic Reviews and Meta-Analyses guidelines [26,27]. The review was registered on PROSPERO.

### Search strategy

We search of eight databases, including PubMed, MEDLINE, Cochrane Library, EMBASE, Web of Science and Chinese Biomedical, Wanfang, and CNKI, was conducted on waste disposal of sharps outside medical institutions for patients with diabetes. Databases were systematically searched using free-text terms related to ‘disposal of sharps waste’ and ‘diabetes’ were searched to February 2023.

### Selection criteria

We have developed a series of inclusion and exclusion criteria to determine articles related to waste disposal of sharp tools outside medical institutions for diabetes patients. Inclusion criteria are as follows: (1) These articles should be related to the disposal of sharp waste by diabetes patients outside medical institutions. (2) This article should describe the behavior of waste disposal with sharp tools (such as whether they will be thrown into household waste or whether they have been injured by needles). (3) This article should focus on diabetes patients. (4) These articles should be published in peer-reviewed journals or meeting minutes.

### Data extraction

Extract the characteristics included in the study, including author, country, study design, sample, age, duration of diabetes, duration of insulin injection, and sharp waste disposal related situations. The extracted data were analyzed using a comprehensive method of descriptive and narrative analysis. Quality assessment was done by using the Cochrane 5.1.0 system [28] evaluation manual to evaluate the bias risk to include in this systematic review See Table 1.

**Table 1:** Risk of bias assessment in the included literature.

Author / year	Stochastic method	Assignment hidden	Blinding	Result data integrity	Results were reported selectively	Other sources of bias
Mekuria et al.,2016 [29]	Randomization	U	U	Y	Y	Y
Atukorala et al.,2018 [30]	U	U	U	Y	Y	Y
Montoya et al.,2019 [31]	U	U	U	Y	Y	Y
Li et al.,2018 [5]	U	U	U	Y	Y	Y
Corte-Real et al.,2022 [10]	Randomization	U	U	Y	Y	U
Tu et al.,2015 [32]	U	U	U	U	Y	Y
Hasan et al.,2019 [33]	Stratified Sampling	U	U	Y	Y	Y
Hasan et al.,2019 [34]	Computer sampling	U	U	Y	Y	U
Choo et al.,2022 [35]	U	U	U	Y	Y	Y
Ishtiaq et al.,2012 [36]	U	U	U	Y	Y	Y
Khan et al.,2021 [37]	U	U	U	Y	Y	Y
Catic et al.,2020 [38]	U	U	U	Y	Y	Y

Note: U = unclear; Y = yes; N = no.

## Results

### Characteristics of the included studies

In 12 identified articles, there are 4155 patients with diabetes, as per Fig 1. These articles were published between 2012 and 2022, with sample sizes ranging from 132 to 1436, and included eleven cross-sectional studies [5,10,29–33,35–38], and one RCT studies [34]. One studies were conducted in Ethiopia [29], one in the Australia [30], two in the United States [5,31], one in Portugal [10], one in China [32], three in Malaysia [33–35], one in Pakistan [36], one in Saudi Arabia [37], and the other in Bosnia and Herzegovina [38]. Twelve studies reported the duration of diabetes in diabetes patients [5,10,30–38], and ten reported the time of insulin injection [10,29–30,32–38]. The summary characteristics of these studies are shown in Table 2.

**Table 2:** Characteristics of the studies included.

Author	Country	Study Design	Sample Size	Age (M/range)	Duration of diabetes (Year)	Duration of insulin injection (Year)	Variables associated with disposal of sharps waste	Sharp waste disposal related situations
Mekuria et al.,2016 [29]	Ethiopia	Cross-sectional	210	43.9	-	<5: 83(39.5%)	Residence, education level and degree of willingness to accept doctor’s advice	49.5% of participants lack knowledge of safe insulin injection waste disposal; 80.7% of respondents had poor behavior; 64.3% of respondents did not insert insulin needles and bayonets into household waste, and 31% of respondents would throw sharp tools on the street when traveling.
Atukorala et al.,2018 [30]	Australia	Cross-sectional	158	53.9	7.4±5.09	3.16±3.88	Education level	41.7% of participants usually put their used sharp tools in the household trash can, 27% of participants involved others (such as family members) when handling sharp tools, and 93% did not receive information on how to handle sharp tools after use.
Montoya et al.,2019 [31]	United States	Cross-sectional	150	56	20±13	-	Duration of diabetes	38% of respondents unsafe discarded the Lancet, 33% unsafe discarded insulin needles, usually throwing these things into household waste; 75% of respondents dispose of insulin pens, vials, ink cartridges, insulin pump supplies, and continuous glucose monitoring sensors in household waste; 64% stated that they have not received education on safe tool disposal practices, and 84% have never visited their municipal website to obtain information about sharps waste disposal.
Li et al.,2018 [5]	United States	Cross-sectional	151	50–64	>20: 104(69%)	-	Duration of diabetes	59% of people handle sharp tools correctly; 34% of patients stated that they have not received formal training on how to handle sharp tools; 70% claim to be very confident or somewhat confident in the correct handling method; Nine patients reported acupuncture injuries in their families; There were 5 cases where they received appropriate training in handling sharp tools.
Corte-Real et al.,2022 [10]	Portugal	Cross-sectional	1436	>18	≥10: 754(52.5%)	<5: 111(8.0%)	-	Most diabetes patients dispose of biological hazardous substances in unclassified household waste (68.1% needle/needle devices, 71.6% bayonets). Approximately 19.0% of patients report that they have been provided with information on appropriate medical tool disposal by healthcare professionals (i.e. medical, nursing, or pharmacy staff).Six patients with diabetes were accidentally stung by needles or willow needles/equipment that had been used or discarded (accounting for 0.8% of medical sharp tool users)
Tu et al.,2015 [32]	China	Cross-sectional	271	63	≥10: 193(71.2%)	<5: 111(41.0%)	-	10.3% of participants handled insulin needles used at home in a safe manner, and 14.8% of participants had previously received guidance on sharp tool handling; 69.7% of participants hope to obtain verbal information about the disposal of sharp tools.
Hasan et al.,2019 [33]	Malaysia	Cross-sectional	304	57.01	≥5: 244(80.3%)	<5: 235(77.3%)	Advice from health care providers on rapid waste disposal, knowledge scores, and diabetes duration	Only 11.5% of people send their used sharp tools to medical institutions for disposal; 35.9% of respondents recalled that after receiving insulin treatment, doctors had advised them to vigorously use and handle it; 11.5% of patients managed to dispose of the sharp tools they had used in healthcare institutions or hospitals.
Hasan et al.,2019 [34]	Malaysia	RCT	Control: 68 Intervene: 68	56.88	≥5: 111(81.6%)	<5: 105(77.2%)	Knowledge and Practice of Sharp Handling	About a quarter of the participants in both groups received advice on sharp disposal from their healthcare providers. About a quarter of people also admit to having previously caused needle injuries to themselves or other family members. Only 1.5% of participants in each group adopted appropriate community sharp handling methods, which collected sharp tools in suitable containers and sent them back to healthcare institutions for final treatment.
Choo et al.,2022 [35]	Malaysia	Cross-sectional	312	56.57	11.77±8.18	3.0	-	The average score of the subjects’ sharp handling knowledge level is 5.90 ± 1.84; Most (46.15%) of the participants held a positive attitude, but 25.30% of the participants held a neutral attitude towards sharp handling; 75% of the participants mishandled the sharp tool and threw the insulin needle directly into the trash can.
Ishtiaq et al.,2012 [36]	Pakistan	Cross-sectional	375	50.6	12.3±7.3	4.4±4.3	Receiving medical education and ability to read and write English	About half of the patients (n = 185) reported that they had been educated by doctors about handling sharp tools.
Khan et al.,2021 [37]	Saudi Arabia	Cross-sectional	406	53.2	13.6±8.7	8.9±7.6	Duration of diabetes	52.5%, 51.7%, and 47.0% of participants were considered to have a high level of knowledge, attitude, and good practical skills, respectively. 78.8% of participants stated that they dispose of their waste in public household garbage bags, and one-third of them do not agree with their safe collection and bringing used waste to hospitals.
Catic et al.,2020 [38]	Bosnia and Herzegovina	Cross-sectional	250	-	≥10: 63(25.2%)	<3: 93(36.9%)	-	67.6% and 66% of patients did not receive guidance on the correct handling of used pens or needles; 48% of respondents put used needles in special boxes, while 44% of respondents throw them into public waste; Those who use special boxes to handle old needles use plastic or glass bottles (34%), plastic bags (23.2%), and the rest use metal or cartoon boxes or simply wrap them on paper.

Note: -: NR.

### Influencing factors of sharp weapon treatment in diabetes patients

#### Impact of social and demographic factors

We explored studies on the management of sharps waste outside of healthcare facilities in diabetic patients from different perspectives. Overall, according to the literature review, sharps waste disposal did not vary by age, gender, glycosylated hemoglobin level, body mass index, race/ethnicity, or the number of insulin needles used [5,31]. Place of residence, education, duration of diabetes and English literacy had a significant effect on appropriate sharp waste disposal. In particular, people with longer duration of diabetes were more likely to engage in inappropriate sharp disposal behaviors, with studies showing that the lowest rate of correct disposal (36%) was reached at diabetes diagnosis more than 30 years ago, and that type I diabetes was better able to dispose of sharp waste than type II diabetes, which may also be related to the duration of diabetes [5,33,37].

#### Other variables

The extent to which willingness to accept physician recommendations from health care providers regarding rapid waste disposal, knowledge and practice of sharps waste disposal had a significant relationship with sharps waste disposal ability in patients with diabetes [29,33,34,36].

### Status of sharps waste disposal for diabetics

#### Knowledge of sharps waste disposal

The results of the study indicate that knowledge of sharps waste disposal is low among people with diabetes [36–38], with Choo results showing that the mean level of sharps disposal knowledge among participants was 5.90 ± 1.84 [35]. Most people with diabetes had not been educated about safe tool disposal practices or instructed on proper handling of insulin pens or needles, and Corte-Real indicated that approximately 19.0% of patients reported that health care professionals (i.e., medical, nursing, or pharmacy staff) provided them with information about proper medical tool disposal [10], and Hasan results showed that only about one-fourth of participants in both groups received advice on sharp disposal from their health care provider [34].

#### Practice of sharps waste disposal

The majority of patients reported that they had no formal training in sharps waste disposal [5,32], and 80.7% of respondents had poor behavior [30]. The few respondents who had received proper sharps waste disposal would put sharps waste in plastic or glass bottles before sending it to a healthcare facility for disposal [33,38]. Other incorrect methods used to dispose of sharps were measures such as throwing them into the street, toilet pits, household dumps, and public waste [10,35,37]. Three studies have reported needle stick injuries to patients or their families as a result of incorrect sharps waste handling [5,33,35].

#### Attitude of sharps waste disposal

The majority of participants had positive attitudes toward sharps waste disposal, with Li’s results showing that 70% of participants claimed to be very or somewhat confident in the correct disposal method [5]; Choo showed that the majority (46.15%) of participants had positive attitudes, but 25.30% were neutral toward sharp disposal [35]; Khan showed that 51.7% of subjects were each considered to have a high attitudes [37]. People would also like to receive more verbal information about the aspects of sharps waste disposal or be able to access information by visiting their municipal websites [31,32].

## Discussion

In the early stages of the diabetics, insulin injections and blood glucose monitoring are more cautious [39]. Due to the chronic nature of the disease, diabetics with longer-lasting diabetes may become tired and frustrated by the prolonged use of medications and ongoing self-management, so they may gradually neglect their diabetes self-care [34,40]. Over time, people with diabetes may progress to more severe disease with a variety of complications. Thus, a longer duration of diabetes may impair their diabetes-related self-management and self-care activities, including their acutely dispositional behaviors [33].

Some of the known barriers to safe disposal include a lack of information on how and where to dispose, a lack of appropriate advice from healthcare professionals, the mistaken belief that sharps disposal information applies only to illicit drug users, and the potential for patients to use community sharps disposal services that may reveal their diabetic status [13–15]. To resolve this paradox, it is necessary to improve the correct rate of sharps waste disposal before and during the use of sharps in diabetic patients.

### Diabetic patients before using the relevant sharps

The fundamental step can be to provide professional training for health care professionals. At present, nursing staff have insufficient knowledge about the safe disposal of out-of-hospital sharps waste and lack relevant knowledge reserves [41]. Hospital managers should regularly train nursing staff on sharps waste management classification [10], disposal and related legal and regulatory knowledge, only when medical staff’s cognitive level is improved and their importance is increased can they play a good guiding role for patients.

Simultaneously, we can provide a variety of educational methods and content. Hasan implemented a health education based on the health belief model for sharps waste disposal for community diabetic patients in the form of lectures [34], demonstration guidance and distribution of pamphlets. The level of sharps waste disposal knowledge in the intervention group was significantly improved through an educational approach to medical sharps for diabetes treatment and the proper handling of sharps prior to disposal [34,35]. Future training for diabetic nurses on sharps disposal outside of medical institutions and incorporating education on safe sharps disposal into daily health education for diabetic patients; peer education, lectures, home visits by community nurses or internet sites to enhance knowledge and methods of safe sharps disposal for diabetic patients in outpatient clinics, wards and communities [10,39].

### Diabetic patients in the process of using the relevant sharps

Several studies have shown that patients are injured by needlesticks when using sharps, so it is important to use safe instruments and sharps containers [5,10,33]. When the sharps container is full, the nurse should instruct the patient to seal the sharps container with a warning sign such as "sharps waste" and then place the container in a hazardous waste bin or send it to a nearby medical facility for disposal. When patients do not have a dedicated sharps container, they can be instructed to use a glass container so they can safely collect and dispose of sharps waste [17,30]. Government environmental departments or community health centers should give patients appropriate incentives in the early stages of promoting standardized disposal of household sharps waste [32,41], such as exchanging new and used needles, free sharps collection boxes, and free blood glucose monitoring, which can motivate more patients to collect and dispose of household sharps waste in a conscious and regulated manner. The government should establish convenient household sharps waste collection terminals: household sharps waste collection sites should be set up in public places such as communities, streets, parks, and shopping centers [31].

## Limitations

There are several limitations of such systematic overviews. On the one hand is the search source, although we tried to improve our search strategy by searching multiple databases using relevant keywords and consulting academic librarians, we inevitably missed some literature, especially relevant research conference proceedings published by non-governmental organizations. On the other hand, as far as the analytical methods of literature search are concerned, this study did not conduct a comparative temporal analysis of the literature across time, and to some extent, it could not fully reveal the technological and sociocultural influences on the behavior of sharps waste disposal in diabetics. In the future, the introduction of knowledge mapping could be considered to map the topics of the documents at different stages.

## Conclusion

There is less research on sharps waste disposal practices in the home setting and incorrect disposal practices increase the risk of needle stick injuries and infections in the community. Our study points out that currently diabetic patients have positive attitudes toward sharps waste disposal, but there is a lack of knowledge and practice of sharps waste disposal and longer duration diabetics are more likely to adopt inappropriate sharps disposal behaviors. Therefore, appropriate measures need to be taken before and during sharps use to improve the rate of proper sharps waste disposal by providing professional training for health care professionals, providing diverse training modalities for diabetic patients, and providing safe sharps boxes, in addition to the need to focus on patients with longer duration of disease in future clinical practice.

## Supporting information

S1 Checklist Preferred Reporting Items for Systematic reviews and Meta-Analyses extension for Scoping Reviews (PRISMA) checklist. (DOC)

## References

[1] World Health Organization (WHO). Diabetes. Available: https://www.who.int/health-topics/diabetes#tab=tab_1 [Accessed 14 May 2023].

[2] World Health Organization, “Status of health-care waste management in selected countries of the Western Pacific Region,” 2015, http://apps.who.int/iris/bitstream/10665/208230/1/9789290617228eng.pdf.

[3] Hosseini Z, Whiting SJ, Vatanparast H. Type 2 diabetes prevalence among Canadian adults—dietary habits and sociodemographic risk factors. Appl Physiol Nutr Metab. 2019 Oct;44(10):1099–1104. doi: 10.1139/apnm-2018-0567 Epub 2019 Aug 6. . 31386561

[4] Catic T, Gojak R, Djekic D. Disposal of Used Pens and Needles from Diabetes Patients Perspective. Mater Sociomed. 2020 Dec;32(4):267–270. doi: 10.5455/msm.2020.32.267-270 . 33628128 PMC7879436

[5] Christ-Crain M, Winzeler B, Refardt J. Diagnosis and management of diabetes insipidus for the internist: an update. J Intern Med. 2021 Jul;290(1):73–87. doi: 10.1111/joim.13261 Epub 2021 Mar 13. . 33713498

[6] Huang L, Katsnelson S, Yang J, Argyrou C, Charitou MM. Factors Contributing to Appropriate Sharps Disposal in the Community Among Patients With Diabetes. Diabetes Spectr. 2018 May;31(2):155–158. doi: 10.2337/ds17-0033 ; PMCID: PMC5951232. 29773935 PMC5951232

[7] Thompson BM, Cook CB. Unsafe Sharps Disposal Among Insulin-Using Patients With Diabetes Mellitus: An Emerging Global Crisis. J Diabetes Sci Technol. 2022 Nov;16(6):1376–1380. doi: 10.1177/19322968211059851 Epub 2021 Dec 1. ; PMCID: PMC9631533. 34852676 PMC9631533

[8] Charbonneau MS, Parsons KA, Danckert DC, Silveira K, Shcherbakova N, Capoccia KL. Sharps Disposal Practices Among People With Diabetes in a Community Care Clinic. Diabetes Spectr. 2022 Fall;35(4):476–483. doi: 10.2337/ds21-0106 Epub 2022 Jun 14. ; PMCID: PMC9668714. 36561648 PMC9668714

[9] Govender D, Ross A. Sharps disposal practices among diabetic patients using insulin. S Afr Med J. 2012 Feb 23;102(3 Pt 1):163–4. doi: 10.7196/samj.5085 . 22380912

[10] Hangulu L, Akintola O. Health care waste management in community-based care: experiences of community health workers in low resource communities in South Africa. BMC Public Health. 2017 May 15;17(1):448. doi: 10.1186/s12889-017-4378-5 ; PMCID: PMC5432984. 28506258 PMC5432984

[11] Costello J, Parikh A. The sticking point: diabetic sharps disposal practices in the community. J Gen Intern Med. 2013 Jul;28(7):868–9. doi: 10.1007/s11606-013-2350-3 ; PMCID: PMC3682034. 23377844 PMC3682034

[12] Olowokure B, Duggal H, Armitage L. The disposal of used sharps by diabetic patients living at home. Int J Environ Health Res. 2003 Jun;13(2):117–23. doi: 10.1080/0960312031000098044 . 12745334

[13] Burke HM, Packer C, Wando L, Wandiembe SP, Muwereza N, Pradhan S, et al. Adolescent and covert family planning users’ experiences self-injecting contraception in Uganda and Malawi: implications for waste disposal of subcutaneous depot medroxyprogesterone acetate. Reprod Health. 2020 Aug 3;17(1):117. doi: 10.1186/s12978-020-00964-1 ; PMCID: PMC7396890. 32746860 PMC7396890

[14] Chalupka SM, Markkanen P, Galligan C, Quinn M. Sharps injuries and bloodborne pathogen exposures in home health care. AAOHN J. 2008 Jan;56(1):15–29; quiz 31–2. doi: 10.3928/08910162-20080101-02 . 18293597

[15] Cooley C, Gabriel J. Reducing the risks of sharps injuries in health professionals. Nurs Times. 2004 Jun 29-Jul 6;100(26):28–9. . 15318686

[16] Corte-Real AL, Duarte LL, Teixeira AL, Cunha MV, Rebelo CC, Azevedo AC, et al. Medical sharps in Portugal: a cross-sectional survey of disposal practices among the diabetic population. BMJ Open. 2022 Sep 23;12(9):e060262. doi: 10.1136/bmjopen-2021-060262 ; PMCID: PMC9511587. 36153023 PMC9511587

[17] Kevitt F, Hayes B. Sharps injuries in a teaching hospital: changes over a decade. Occup Med (Lond). 2015 Mar;65(2):135–8. doi: 10.1093/occmed/kqu182 Epub 2014 Dec 29. . 25548258

[18] Montoya JM, Thompson BM, Boyle ME, Leighton ME, Cook CB. Patterns of Sharps Handling and Disposal Among Insulin-Using Patients With Diabetes Mellitus. J Diabetes Sci Technol. 2021 Jan;15(1):60–66. doi: 10.1177/1932296819882926 Epub 2019 Oct 22. ; PMCID: PMC7782998. 31640410 PMC7782998

[19] Majumdar A, Sahoo J, Roy G, Kamalanathan S. Improper sharp disposal practices among diabetes patients in home care settings: Need for concern? Indian J Endocrinol Metab. 2015 May-Jun;19(3):420–5. doi: 10.4103/2230-8210.152792 ; PMCID: PMC4366785. 25932402 PMC4366785

[20] Hassan NM, Shalaby SES, Atalla AO, Younis EA. Toward safe environment: injection device disposal among diabetic patients attending tertiary care academic clinic in Middle Delta, Egypt. Environ Sci Pollut Res Int. 2021 May;28(18):23193–23203. doi: 10.1007/s11356-021-12393-z Epub 2021 Jan 13. . 33442798

[21] Weltman AC, Short LJ, Mendelson MH, Lilienfeld DE, Rodriguez M. Disposal-related sharps injuries at a New York City Teaching Hospital. Infect Control Hosp Epidemiol. 1995 May;16(5):268–74. doi: 10.1086/647106 . 7657974

[22] Moray KV, Manjunath K, Martina Shalini AJ, Pricilla S RA, John SM, Prasad JH. The insulin sharps disposal study: Evaluation of a structured patient education initiative in an urban community health centre in India. J Family Med Prim Care. 2020 Dec 31;9(12):6164–6170. doi: 10.4103/jfmpc.jfmpc_1295_20 ; PMCID: PMC7928141. 33681058 PMC7928141

[23] Olowokure B, Duggal H, Armitage L. The disposal of used sharps by diabetic patients living at home. Int J Environ Health Res. 2003 Jun;13(2):117–23. doi: 10.1080/0960312031000098044 . 12745334

[24] Macalino GE, Springer KW, Rahman ZS, Vlahov D, Jones TS. Community-based programs for safe disposal of used needles and syringes. J Acquir Immune Defic Syndr Hum Retrovirol. 1998;18 Suppl 1:S111–9. doi: 10.1097/00042560-199802001-00019 . 9663633

[25] Hanrahan A, Reutter L. A critical review of the literature on sharps injuries: epidemiology, management of exposures and prevention. J Adv Nurs. 1997 Jan;25(1):144–54. doi: 10.1046/j.1365-2648.1997.1997025144.x . 9004023

[26] Page MJ, McKenzie JE, Bossuyt PM, Boutron I, Hoffmann TC, Mulrow CD, et al. The PRISMA 2020 statement: an updated guideline for reporting systematic reviews. BMJ. 2021 Mar 29;372:n71. doi: 10.1136/bmj.n71 ; PMCID: PMC8005924. 33782057 PMC8005924

[27] Rostom A, Dubé C, Cranney A, Saloojee N, Sy R, Garritty C, et al. Celiac disease. Evid Rep Technol Assess (Summ). 2004 Jun;(104):1–6. ; PMCID: PMC4781297. 15346868 PMC4781297

[28] Li Y, Xing X, Shi X, Yan P, Chen Y, Li M, et al. The effectiveness of music therapy for patients with cancer: A systematic review and meta-analysis. J Adv Nurs. 2020 May;76(5):1111–1123. doi: 10.1111/jan.14313 Epub 2020 Feb 19. . 32017183

[29] Basazn Mekuria A, Melaku Gebresillassie B, Asfaw Erku D, Taye Haile K, Melese Birru E. Knowledge and Self-Reported Practice of Insulin Injection Device Disposal among Diabetes Patients in Gondar Town, Ethiopia: A Cross-Sectional Study. J Diabetes Res. 2016;2016:1897517. doi: 10.1155/2016/1897517 Epub 2016 Sep 25. ; PMCID: PMC5055957. 27738637 PMC5055957

[30] Atukorala KR, Wickramasinghe SI, Sumanasekera RDN, Wickramasinghe KH. Practices related to sharps disposal among diabetic patients in Sri Lanka. Asia Pac Fam Med. 2018 Dec 7;17:12. doi: 10.1186/s12930-018-0049-7 ; PMCID: PMC6286594. 30555274 PMC6286594

[31] Montoya JM, Thompson BM, Boyle ME, Leighton ME, Cook CB. Patterns of Sharps Handling and Disposal Among Insulin-Using Patients With Diabetes Mellitus. J Diabetes Sci Technol. 2021 Jan;15(1):60–66. doi: 10.1177/1932296819882926 Epub 2019 Oct 22. ; PMCID: PMC7782998. 31640410 PMC7782998

[32] Tu H, Lu X, Wang J, Sheng Z, Liu D, Li J, et al. At-home disposal practices of used insulin needles among patients with diabetes in China: A single-center, cross-sectional study. Front Public Health. 2022 Dec 8;10:1027514. doi: 10.3389/fpubh.2022.1027514 ; PMCID: PMC9772984. 36568796 PMC9772984

[33] Hasan UA, Mohd Hairon S, Yaacob NM, Daud A, Abdul Hamid A, Hassan N, et al. Factors Contributing to Sharp Waste Disposal at Health Care Facility Among Diabetic Patients in North-East Peninsular Malaysia. Int J Environ Res Public Health. 2019 Jun 26;16(13):2251. doi: 10.3390/ijerph16132251 ; PMCID: PMC6651231. 31247892 PMC6651231

[34] Hasan UA, Mohd Hairon S, Yaacob NM, Daud A, Abdul Hamid A, Hassan N, et al. Effectiveness of Diabetes Community Sharp Disposal Education Module in Primary Care: An Experimental Study in North-East Peninsular Malaysia. Int J Environ Res Public Health. 2019 Sep 11;16(18):3356. doi: 10.3390/ijerph16183356 ; PMCID: PMC6765895. 31514391 PMC6765895

[35] Choo JY, Ng YP, Ariffin Abdul Jamil AK, Heng WK, Ng YM, Ng J, et al. An exploratory study on the knowledge, attitude and practice of sharp disposal among type 2 diabetes mellitus patients in Northern Peninsular Malaysia. Diabetes Metab Syndr. 2022 Apr;16(4):102479. doi: 10.1016/j.dsx.2022.102479 Epub 2022 Apr 10. . 35427913

[36] Ishtiaq O, Qadri AM, Mehar S, Gondal GM, Iqbal T, Ali S, et al. Disposal of syringes, needles, and lancets used by diabetic patients in Pakistan. J Infect Public Health. 2012 Apr;5(2):182–8. doi: 10.1016/j.jiph.2012.02.002 Epub 2012 Mar 30. . 22541266

[37] Khan AM, Al Ghamdi RA, Alswat KA. Knowledge, Attitude and Practice on Disposal of Sharps Waste at Home Among Patients with Diabetes and their Caregivers. Curr Diabetes Rev. 2021;17(5):e170920186033. doi: 10.2174/1573399816999200917162514 . 32942978

[38] Catic T, Gojak R, Djekic D. Disposal of Used Pens and Needles from Diabetes Patients Perspective. Mater Sociomed. 2020 Dec;32(4):267–270. doi: 10.5455/msm.2020.32.267-270 ; PMCID: PMC7879436. 33628128 PMC7879436

[39] Tomkins M, Lawless S, Martin-Grace J, Sherlock M, Thompson CJ. Diagnosis and Management of Central Diabetes Insipidus in Adults. J Clin Endocrinol Metab. 2022 Sep 28;107(10):2701–2715. doi: 10.1210/clinem/dgac381 ; PMCID: PMC9516129. 35771962 PMC9516129

[40] Patti G, Ibba A, Morana G, Napoli F, Fava D, di Iorgi N, et al. Central diabetes insipidus in children: Diagnosis and management. Best Pract Res Clin Endocrinol Metab. 2020 Sep;34(5):101440. doi: 10.1016/j.beem.2020.101440 Epub 2020 Jun 29. . 32646670

[41] Morbach S, Lobmann R, Eckhard M, Müller E, Reike H, Risse A, et al. Diabetic Foot Syndrome. Exp Clin Endocrinol Diabetes. 2021 Aug;129(S 01):S82–S90. doi: 10.1055/a-1284-6412 Epub 2020 Dec 22. . 33352597

